# Mapping of equine mesenchymal stromal cell surface proteomes for identification of specific markers using proteomics and gene expression analysis: an in vitro cross-sectional study

**DOI:** 10.1186/s13287-018-1041-8

**Published:** 2018-10-25

**Authors:** Louise Bundgaard, Allan Stensballe, Kirstine Juul Elbæk, Lise Charlotte Berg

**Affiliations:** 1 Department of Veterinary Clinical Sciences, University of Copenhagen, Agrovej 8, DK-2630 Taastrup, Denmark; 2 Department of Health Science and Technology, Aalborg University, Fredrik Bajers Vej 7, 9220 Aalborg Ø, Denmark

**Keywords:** Mesenchymal stromal cells, CD surface markers, Biotinylation, Mass spectrometry, Gene expression, Equine

## Abstract

**Background:**

Stem cells have great potential for tissue regeneration, but before stem cell populations can be used in the clinic, it is crucial that the stem cells have been definitely characterized by a set of specific markers. Although there have been attempts to identify a set of immunophenotypic markers to characterize equine mesenchymal stromal cells (MSCs), immunophenotyping of equine MSCs is still challenging due to the limited availability of suitable antibodies of high quality and consistent performance across different laboratories. The aim of this study was to evaluate a strategy for mapping the equine MSCs surface proteome by use of biotin-enrichment and mass spectrometry (MS) analysis and mine the proteome for potential equine MSCs surface markers belonging to the cluster of differentiation protein group. Gene expression analysis was used for verification.

**Methods:**

Equine MSCs derived from bone marrow (BM) (*n* = 3) and adipose tissue (AT) (*n* = 3) were expanded to P3 and either used for (1) cell differentiation into mesodermal lineages (chondrogenic and osteogenic), (2) enrichment of the MSCs surface proteins by biotinylation followed by in-gel digest of the isolated proteins and nanoLC-MS/MS analysis to unravel the enriched cell surface proteome, and (3) RNA isolation and quantitative real-time reverse transcriptase PCR analysis of the CD29, CD44, CD90, CD105, CD166, CD34, CD45, and CD79a gene expression.

**Results:**

A total of 1239 proteins at 1% FDR were identified by MS analysis of the enriched MSCs surface protein samples. Of these proteins, 939 were identified in all six biological samples. The identified proteins included 19 proteins appointed to the cluster of differentiation classification system as potential cell surface targets. The protein and gene expression pattern was positive for the commonly used positive MSCs markers CD29, CD44, CD90, CD105, and CD166, and lacked the negative MSCs markers CD34, CD45, and CD79a.

**Conclusions:**

The findings of this study show that enrichment of the MSCs surface proteome by biotinylation followed by MS analysis is a valuable alternative to immunophenotyping of surface markers, when suitable antibodies are not available. Further, they support gene expression analysis as a valuable control analysis to verify the data.

## Background

Similar to humans, the horse is a long-lived athletic species, and regenerative medicine is seen as the next-generation treatment to restore normal function of cells and tissues damaged by time, injury or disease. Stem cells have great potential for tissue repair and regeneration; that is why, they are intensely investigated in equine clinical research [1, 2]. However, before any type of stem cell can be applied in practice, it is crucial that the isolated stem cells have been definitively characterized by a set of specific functional or phenotypic markers.

A common definition of equine mesenchymal stromal cells (MSCs) is still lacking, but in humans, characterization of these cells is well defined by the criteria of the International Society for Cellular Therapy (ISCT) [3]. ISCT has stated that MSCs must be plastic adherent and be capable of differentiating toward the osteogenic, chondrogenic, and adipogenic lineage. Furthermore, they must express CD29, CD44, CD73, CD90, and CD105 and lack expression of CD14, CD34, CD45, CD79a, and MHC II. Although there have been attempts to identify a set of immunophenotypic markers to characterize equine MSCs [4–6], immunophenotyping of equine MSCs is still challenging due to the limited availability of suitable antibodies of high quality and consistent performance across different laboratories [5, 7]. Radcliffe et al. [5] found that only 4 of 15 antibodies tested (27%) were reactive to equine molecules using flow cytometry analysis. De Schauwer et al. [7] found that 11 out of 30 antibodies (37%) were reactive to equine molecules, and they were not able to confirm cross-reactivity of two tested clones used in other studies to characterize equine MSCs. In general, cross-reactivity for antihuman monoclonal antibodies to equine epitopes is limited. In a large test of 379 antihuman monoclonal antibodies, only 14 recognized the corresponding epitopes on isolated equine leukocytes, which is less than 5% [8]. This illustrates the need for studies evaluating alternative methods for identification of MSCs markers.

Mass spectrometry (MS) is a rapidly advancing technology for identification and quantification of proteins applicable to stem cell investigation [9], thus circumventing the need of antibodies for cell phenotyping. This analysis technology in combination with cell surface protein biotinylation to enrich the plasma membrane proteins has successfully been used for comprehensive analysis of cell surface proteomes, e.g., to identify spermatozoa surface proteins and quantitative changes during epididymal maturation in boars [10] and bulls [11], and to identify surface proteins of human dental pulp stem cells and their variation between culture conditions [12]. Furthermore, this approach has been used for comprehensive analysis of the human BM-MSCs surface proteome [13, 14].

Gene expression analysis at the mRNA level is another potentially valuable alternative to antibody-based methods, and compared to MS, which is still only accessible at specialized proteomic core facilities, qPCR analysis is available in most laboratories.

The aim of this study was to analyze and compare the equine adipose tissue (AT)-derived and bone marrow (BM)-derived MSCs surface proteome by use of biotin enrichment and MS analysis and mine the proteomes for potential equine MSCs surface markers appointed to the cluster of differentiation (CD) classification system. Gene expression analysis was used to verify the results.

## Methods

The experimental protocol was approved by the Ethics and Welfare Committee of Department of Veterinary Clinical Sciences, University of Copenhagen, Denmark.

### Animals used in the study

MSCs derived from BM (*n* = 3) and AT (*n* = 3) were obtained from mares of mixed breeds, weighing approx. 500 kg and ranging from 13 to 17 years. The horses were euthanized with captive bolt and exsanguination at the Large Animal Teaching Hospital, University of Copenhagen, for unrelated reasons.

### Sampling of bone marrow and adipose tissue

BM-MSCs were isolated from sternal BM aspirates. The skin over the sternum was surgically prepared immediately post-euthanasia in a routine manner, and 5–7 mL BM was aspirated using a Jamshidi biopsy needle (11 gauge, 12.7 cm) (Stryker, Kalamazoo, MI, USA) in a 20-mL syringe preloaded with 1 mL 10% 0.109 M trisodium citrate. The aspirate was transferred to a 50-mL falcon tube and inverted gently.

AT-MSCs were isolated from adipose tissue from the region above the biceps femoris muscle at the base of the tail. The skin was surgically prepared immediately post-euthanasia in a routine manner, and a 10 cm × 10 cm piece of epidermis and dermis was removed to expose the subcutaneous AT. Approximately 15 g of AT was harvested, avoiding fascia and blood vessels and transferred to a 50-mL falcon tube with 30 mL sterile PBS.

All samples were transported at room temperature to the laboratory immediately and further processed within ~ 1 h after sampling.

### Isolation of mesenchymal stromal cells

BM was layered gently onto Histopaque-1077 (Sigma-Aldrich, St. Louis, MI, USA) 1:1 (*v*/*v*) and centrifuged 30 min at 600*g*. The buffy layer containing the mononuclear cells was recovered and washed twice in sterile PBS (5 min at 200*g*), supernatant was discarded, and the cell pellet resuspended in 24 mL expansion medium (EM) (Dulbecco’s modified Eagle’s medium (DMEM) 1 g/L glucose, with phenol red, GlutaMAX, and pyruvate (Thermo Fischer Scientific, Waltham, MA, USA) supplemented with 10% (*v*/*v*) fetal bovine serum (FBS) (Thermo Fischer Scientific, Waltham, MA, USA), 100 U/mL penicillin and 100 μg/mL streptomycin (Thermo Fischer Scientific, Waltham, MA, USA), and 25 μg/mL amphotericin B (Thermo Fischer Scientific, Waltham, MA, USA)). The cells were distributed equally in two T75 cm^2^ culture flasks and cultured at 37 °C in a humidified atmosphere containing 5% CO_2_.

AT was washed three times in sterile PBS, dissected to smaller pieces, and digested in sterile filtered (0.2 μm) (Sartorius, Goettingen, Germany) enzyme medium (DMEM 1 g glucose/L, with phenol red, GlutaMAX, and pyruvate, supplemented with 100 U/mL penicillin and 100 μg/mL streptomycin, 50 μg/mL gentamycin (Sigma-Aldrich, St. Louis, MI, USA), and 1 mg/mL collagenase type I (Thermo Fischer Scientific, Waltham, MA, USA)) for 3 h at 37 °C and 30 rpm. Released cells were isolated through a cell strainer (70 μm) (Thermo Fischer Scientific, Waltham, MA, USA), rinsed twice in sterile PBS, supernatant discarded, and the cell pellet resuspended in 24 mL EM supplemented with 50 μg/mL gentamycin. The cells were distributed equally in two T75 cm^2^ culture flasks and cultured at 37 °C in a humidified atmosphere containing 5% CO_2_.

After overnight incubation, non-adherent cells were removed and fresh medium added. Medium was changed every 2–3 days. Cells were passaged (P1) at ~ 80% confluency (10–12 days) using trypsin-EDTA (0.25%) (Thermo Fischer Scientific, Waltham, MA, USA), replating ratio 1:3. P1 cells were further expanded in EM without amphotericin B, supplemented with 50 μg/mL gentamycin (only AT-MSCs). At 80% confluency (8–10 days), cells were detached with trypsin-EDTA, rinsed in sterile PBS, counted, and cryopreserved in FBS supplemented with 10% dimethyl sulfoxide (Sigma-Aldrich, St. Louis, MI, USA) in 1–2 × 10^6^ aliquots. Cryopreserved MSCs (~ 2 × 10^6^ cells) were thawed (P2) and expanded in two T75 cm^2^ flasks in EM without amphotericin B, supplemented with 50 μg/mL gentamycin (only AT-MSCs). At ~ 90% confluency (10–12 days), the cells were passaged using trypsin-EDTA (0.25%) and replated in ratio 1:3 (P3). P3 cells at ~ 90% confluency (5–6 days) were either used for cell differentiation into mesodermal lineages (chondrogenic and osteogenic), biotinylation of cell surface proteins, or RNA isolation.

### Cell differentiation into mesodermal lineages

Cells were passaged (P4) to 48-well plates at a density of ~ 10^3^ cells/well (9090 cells/cm^2^) and expanded in EM without amphotericin B, supplemented with 50 μg/mL gentamycin (only AT-MSCs). At ~ 80% confluency, cells were washed with sterile PBS and medium was changed to chondrogenic differentiation medium (DMEM 4.5 g/L glucose, without phenol red, supplemented with 1% (*v*/*v*) GlutaMAX (Thermo Fischer Scientific, Waltham, MA, USA), 1% (*v*/*v*) pyruvate (Thermo Fischer Scientific, Waltham, MA, USA), 2% (*v*/*v*) FBS, 1% (*v*/*v*) insulin-transferrin-selenium (ITS) (Thermo Fischer Scientific, Waltham, MA, USA), 100 U/mL penicillin and 100 μg/mL streptomycin, 10^−7^ M dexamethasone (Sigma-Aldrich, St. Louis, MI, USA), 50 μg/mL ascorbic acid (l-ascorbic acid 2-phosphate) (Sigma-Aldrich, St. Louis, MI, USA), 10 ng/mL TGF-ß3 (recombinant human TGF-ß3) (R&D Systems, Inc., Minneapolis, MI, USA), and 50 μg/mL gentamycin (only AT-MSCs)) or osteogenic differentiation medium (DMEM 1 g/L glucose, without phenol red, supplemented with 1% (*v*/*v*) pyruvate (Thermo Fischer Scientific, Waltham, MA, USA), 2% (*v*/*v*) FBS, 1% (*v*/*v*) ITS, 100 U/mL penicillin and 100 μg/mL streptomycin, 10^−7^ M dexamethasone, 50 μg/mL ascorbic acid (l-ascorbic acid 2-phosphate), 10 μL/mL 1 M ß-glycerophosphate (ß-glycerophosphate, disodium salt, pentahydrate) (Merck KGaA, Darmstadt, Germany), and 50 μg/mL gentamycin (only AT-MSCs). Differentiation medium was changed every 3 days for 21 days. Confirmation of chondrogenic and osteogenic differentiation was performed by staining for proteoglycans in the extracellular matrix using 0.1% Safranin O, pH 4.6 (Merck KGaA, Darmstadt, Germany) and calcified extracellular matrix deposits using 2% Alizarin red staining, pH 4.2 (Sigma-Aldrich, St. Louis, MI, USA), respectively.

### Mesenchymal stromal cell surface biotinylation

Cell surface proteins were biotinylated and isolated according to the manufacturer’s protocol (Pierce cell surface protein isolation kit, https://www.thermofisher.com/order/catalog/product/89881) with few modifications. In short, EM was removed and cells were washed twice in ice-cold PBS and incubated with a biotin labelling solution for 30 min at 4 °C on a rocking platform. The reaction was quenched with a quenching solution and cells were scraped (cell scraper, Corning Incorporated, Corning, NY, USA) into solution, pooled in a 50 mL conical tube, and washed twice with TBS. Cells were resuspended in lysis buffer supplemented with protease inhibitor (complete protease inhibitor cocktail tablets) (Roche, Basel, Switzerland) 25:1 (*v*/*v*), transferred to a 1.5-mL microcentrifuge tube, and incubated on ice 30 min, vortexing every 5 min for 5 s. Cell lysate was centrifuged at 10,000 *g* for 2 min at 4 °C and the supernatant was transferred to a new tube. The cell lysate was applied to a neutravidin agarose gel column, incubated for 60 min at RT on a rocking platform, and then centrifuged for 1 min at 1000*g*. The column was washed with wash buffer supplemented with protease inhibitor (25:1 (*v*/*v*)), and centrifuged for 1 min at 1000 *g*. This step was repeated three times. A 4× Laemmli sample buffer (Bio-Rad Laboratories, Inc., Hercules, CA, USA) with 50 mM dithiothreitol was added to the column, and the reaction was incubated for 60 min at RT on a rocking platform followed by centrifugation for 2 min at 1000 *g*. The eluate was stored at − 80 °C until further processing.

### SDS-PAGE and in-gel digestion of biotinylated cell surface proteins

Samples were thawed on ice and the proteins in 25 μL 4× Laemmli buffer were reduced by heating for 7 min at 95 °C, and further processed by SDS-PAGE (10 well, mini protean TGX gel 4–20%) (Bio-Rad Laboratories, Inc., Hercules, CA, USA) at 180 V for 10 min, to remove lithium dodecyl sulphate from the sample and separate proteins according to molecular weight allowing the proteins to penetrate the resolving gel for approx. 1 cm. After staining with coomassie blue (Sigma-Aldrich, St. Louis, MI, USA), the visible band was excised into small cubes and transferred to individual 1.5 mL microcentrifuge tubes. The gel pieces were washed with 50 mM triethylammonium bicarbonate buffer (TEAB) (Sigma-Aldrich, St. Louis, MI, USA) for 15 min at RT, followed by addition of acetonitrile (Honeywell-Fluka, Morris Plains, NJ, US) in a 1:1 solution with TEAB and incubation for 15 min at RT, removing of all liquids and shrinking of the gel pieces with acetonitrile. These washing steps were repeated four times. Proteins in the gel pieces were reduced and alkylated by incubation with 1.2 μL 0.5 M tris (2-carboxyethyl)phosphine hydrochlorid (Sigma-Aldrich, St. Louis, MI, USA) per 50 μL TEAB and 6 μL 0.5 M chloroacetamide (Sigma-Aldrich, St. Louis, MI, USA) per 50 μL TEAB for 30 min at 37 °C followed by a fifth washing sequence. Proteins were digested with 12.5 ng trypsin (Thermo Fischer Scientific, Waltham, MA, USA)/μL TEAB at 37 °C overnight, and the supernatant containing the extracted peptides was transferred to a fresh microcentrifuge tube. The gel pieces were then incubated in 1:20 (*v*/*v*) formic acid (Honeywell-Fluka, Morris Plains, NJ, USA) in TEAB for 15 min at RT, the supernatant added to the peptide solution, the gel pieces incubated in acetonitrile for 15 min at RT, and the supernatant transferred to the peptide solution. The peptides were concentrated in a vacuum centrifuge and stored at − 80 °C until further processing.

### LC-MS/MS analysis

Peptides were resuspended in resuspension buffer (2% acetonitrile, 0.1% triflouroacetic acid (Thermo Fischer Scientific, Waltham, MA, USA), 0.1% formic acid in MilliQ water) and a volume corresponding to ~ 1 μg peptide was analyzed by nanoLC-MS/MS (Thermo Scientific Dionex Ultimate 3000 RSLC) coupled in-line to a Thermo Scientific Q Exactive HF mass spectrometer. The peptide separation was accomplished using a precolumn setup (Acclaim PepMap 100 C18 2 cm 100 μm precolumn; 75 μm 75 cm main column) (Thermo Fischer Scientific, Waltham, MA, USA) and a 35-min gradient from 10% buffer B (99.9% acetonitrile) to 35% buffer B and the buffer A being 99.1% MilliQ with 0.1% formic acid. The mass spectrometer was set to acquire MS1 data from m/z 375–1500 at *R* = 60 k and MS2 at *R* = 30 k allowing up to 20 precursor ions per MS1 scan.

### RNA isolation and quantitative real-time reverse transcriptase PCR analysis

Cells were lysed in TRI Reagent (Molecular Research Center, Inc., Cincinnati, OH, USA) and stored at − 80 °C until further processing. After thawing, the cell lysate was further homogenized through a QIAshredder column (Qiagen, Hilden, Germany) for 2 min at 12,000 *g*. The homogenate was phase separated by adding 0.2 mL chloroform per mL TRI Reagent, shaked vigorously for 15 s allowing it to stand for 15 min at RT, and centrifuged at 12,000 *g* for 15 min at 4 °C. The upper phase containing the RNA was transferred to a fresh tube. The RNA was precipitated by adding 0.5 mL 2-propanol per mL TRI Reagent, incubation for 8 min at RT, followed by centrifugation at 12,000 *g* for 8 min at 4 °C. After removing the supernatant, the RNA pellet was washed by adding 1 mL 75% ethanol per 1 mL TRI Reagent and centrifugation at 7500 *g* for 5 min at 4 °C. The supernatant was removed, and the pellet was air dried for 5–7 min. The pellet was resuspended in distilled water and incubated for 15 min at 60 °C. Total RNA concentration was determined by optical density measurement (NanoDrop TM Spectrophotometer (Thermo Fischer Scientific, Waltham, MA, USA)), and total RNA isolates were kept at − 80 °C until further processing.

cDNA was synthesized from 200 ng total RNA. Reverse transcriptase PCR mastermix (Promega, Madison, WI, USA) consisted of 5 μL RT buffer, 1.3 μL dNTP mix (10 μM) (Thermo Fischer Scientific, Waltham, MA, USA), 0.25 μL random hexamer primers (2 μg/μL) (TAG Copenhagen, Copenhagen, Denmark), 0.25 μL Oligo-dT primers (0.5 μg/μL) (TAG Copenhagen, Copenhagen, Denmark), 0.8 μL RNasin® Plus RNase inhibitor (40 U/μL) (Promega, Madison, WI, USA), 1 μL M-MLV Reverse Transcriptase (200 U/μL) (Promega, Madison, WI, USA), and sterile water. Reverse transcription was performed in a BIOmetra® T-Gradient thermocycler (Thermo Fischer Scientific, Waltham, MA, USA) at 25 °C for 10 min, 42 °C for 60 min, and 95 °C for 5 min. Samples were stored at − 20 °C.

Species-specific intron-spanning equine primers were used to amplify CD29, CD44, CD90, CD105, CD166, CD34, CD45, and CD79a. Primers are listed in Table 1. Primers were purchased from TAG Copenhagen (Copenhagen, Denmark). Quantitative real-time reverse transcriptase PCR was performed in triplicates using the LightCycler® Fast Start DNA Master SYBR Green I and LightCycler® Real-Time PCR System (Roche, Basel, Switzerland). cDNA from equine spleen was used as a positive control.

**Table 1:** Species-specific primers used to amplify specific genes

Primer name	Forward primer 5′-3′	Reverse primer 5′-3′
CD29	GTG AGA TGT GTC AGA CGT GC	AGA ACC AGC AGT CAT CCA CA
CD44	TTC ATA GAA GGG CAC GTG GT	GCC TTT CTT GGT GTA GCG AG
CD90	TCT CCT GCT GAC AGT CTT GC	GGA CCT TGA TGT TGT ACT TGC
CD105	TTC TGG GCC ACT GGT GAA TA	TGC AAT GCA GAC TCG AGA TG
CD166	GCA GAA AAC CAG CTG GAG AG	AGC GAG GAG TAG ACC AAC GA
CD34	CTC CAG CTG TGA GGA CTT TA	AAG TTC TGG ATC CCC ATC CT
CD45	CTC CTC ATT CAC TGC AGA GA	GGT ACT GCT CAA ATG TGG GA
CD79a	AGG GAG CCA CAT CAA CAT CA	CGT TGC CTT CCT TAG CTT GG
18 s	GAT ACC GCA GCT AGG AAT	ATC TGT CAA TCC TGT CCG
ß-actin	CGT GGG CCG CCC TAG GCA CCA	TTG GCC TTA GGG TTC AGG GGG G
GAPDH	GGG TGG AGC CAA AAG GGT CAT CAT	AGC TTT CTC CAG GCG GCA GGT CAG

#### Data analysis

Raw data was searched against the *Equus caballus* reference sequence database from Uniprot (UP000002281; May 16, 2017; 22,698 proteins) using MaxQuant search engines (MaxQuant v.1.6.0.1 and Perseus v.1.6.0.2). Label-free quantification (LFQ) was based on total ion chromatogram normalization [15]. The online database STRING-DB was used to further identify uncharacterized proteins based on gene [16]. Only proteins with at least two unique peptide sequences and FDR < 1% was included. The MS proteomics data have been deposited and made publically available to the ProteomeXchange Consortium via the PRIDE partner repository with the dataset identifier PXD008884 [17].

Relative mRNA expression was calculated using the efficiency corrected calculation method also known as the Roche Applied Sciences E(efficiency)-method: Normalized relative ratio (NRR) = E_t_^CT (target calibrator) – CT (target sample)^/E_r_^CT (reference calibrator) – CT (reference sample)^. All results were normalized to the reference gene gluceraldehyde-3-phosphate dehydrogenase (GAPDH) selected after initial testing of three reference genes (GAPDH, β-actin and ribosomal RNA (18 S)) [18].

## Results

### Cellular morphology and differentiation into mesodermal linages

All cell lines were plastic adherent and exhibited a fibroblast-like morphology. Chondrogenic differentiated cells stained positive for proteoglycans in the extracellular matrix and osteogenic differentiated cells stained positive for calcified extracellular matrix deposits on day 21 after induction of differentiation.

### MS analysis

A total of 1239 proteins were identified with at least two unique peptide sequences and FDR < 1%. Among the identified proteins were a total of 19 proteins appointed to the CD classification system as potential cell surface targets for immunophenotyping of cells (Tables 2 and 3). The CD proteins were identified in all samples, except CD49c and CD228, which were not identified in the samples from AT-MSCs; CD61, which was not identified in one of the samples from AT-MSCs; CD56, which was not identified in any of the BM-MSCs samples; and CD105, which was not identified in one of the samples from BM-MSCs.

**Table 2:** Identified genes and/or proteins appointed to the cluster of differentiation (CD) classification system

Protein	Alternative protein name	Accession number	Gene name	AT-MSCs1	AT-MSCs2	AT-MSCs3	BM-MSCs1	BM-MSCs2	BM-MSCs3
CD29	Integrin subunit beta 1	F6UR46	ITGB1	+/+	+/+	+/+	+/+	+/+	+/+
CD44		Q05078	CD44	+/+	+/+	+/+	+/+	+/+	+/+
CD49a	Integrin subunit alpha 1	F6SHD8	ITGA1	+	+	+	+	+	+
CD49c	Integrin subunit alpha 3	F6W8D8	ITGA3	+	+	+	+	+	+
CD49d	Integrin subunit alpha 4	F6ZZX8	ITGA4	–	–	–	+	+	+
CD49e	Integrin subunit alpha 5	F6QLZ6	ITGA5	+	+	+	+	+	+
CD51	Integrin subunit alpha V	F6W3W7	ITGAV	+	+	+	+	+	+
CD56	Neural cell adhesion molecule 1	F7BT93	NCAM1	+	+	+	–	–	–
CD61	Integrin subunit beta 3	F7A370	ITGB3	+	+	–	+	+	+
CD71	Transferrin receptor 1	Q2V905	TFRC	+	+	+	+	+	+
CD73	Ecto 5′ nucleotidase	F6XEP5	NT5E	+	+	+	+	+	+
CD90	Thy-1 cell surface antigen	F6ZC75	THY1	+/+	+/+	+/+	+/+	+/+	+/+
CD91	Alpha-2-macroglobulin receptor	F6Q221	LRP1	+	+	+	+	+	+
CD105	Endoglin	F6W046	ENG	+/+	+/+	+/+	+/+	−/+	+/+
CD109		F6V1V8	CD109	+	+	+	+	+	+
CD142	Tissue factor	F6UIK6	F3	+	+	+	+	+	+
CD166	Activated leukocyte cell adhesion molecule	F7B5L3	ALCAM	+/+	+/+	+/+	+/+	+/+	+/+
CD228	Melanotransferrin	F7DGD6	MFI2	–	–	–	+	+	+
CD315	Prostaglandin F2 receptor inhibitor	F6WPE1	PTGFRN	+	+	+	+	+	+

Cells are isolated from samples harvested from three different horses (1–3). The cell surface proteins were enriched with biotinylation, and the samples analyzed by mass spectrometry. Raw data was searched against the *Equus caballus* reference sequence database from Uniprot (UP000002281; May 16, 2017; 22,698 proteins) giving information about protein names, accession numbers, and gene names. Gene expression was used to verify the data. (+) indicates that the protein was identified in the sample, (−) indicates that the protein was not identified in the sample. (+/+) indicates identification both on the protein and gene level. (−/+) indicates no identification on the protein level, but identification on the gene level

*AT-MSCs* adipose tissue-derived mesenchymal stromal cells, *BM-MSCs* bone marrow-derived mesenchymal stromal cells

**Table 3:** Cluster of differentiation proteins (CD) identified in equine AT-MSCs and/or equine BM-MSCs and their characteristics in relation to AT-MSCs and BM-MSCs described in the literature

Protein	Gene name	Characteristics	References
Identified cluster of differentiation proteins previously reported as present on the surface of equine MSCs			
CD29	ITGB1	Consistently observed equine MSCs surface protein. Positive human MSCs surface marker according to ISCT	[5, 7, 19, 21]
CD44	CD44	Inconsistently observed equine MSCs surface marker. Positive human MSCs surface marker according to ISCT	[5, 7, 19, 21]
CD51	ITGAV	Identified on the surface of MSCs derived from equine peripheral blood and AT. Human endothelial cell marker. Upregulated in mouse BM-MSCs during osteoblastic differentiation	[20]
CD71	TFRC	Identified on MSCs derived from equine umbilical cord intervascular matrix	[19]
CD73	NT5E	Inconsistently observed equine MSCs surface marker. Positive human MSCs surface marker according to ISCT	[22]
CD90	THY1	Inconsistently observed equine MSCs surface marker. Positive human MSCs surface marker according to ISCT	[5, 7, 19, 21]
CD105	ENG	Inconsistently observed equine MSCs surface marker. Positive human MSCs surface marker according to ISCT	[7, 19, 21]
CD166	ALCAM	Identified on MSCs derived from equine umbilical cord intervascular matrix	[19]
Identified cluster of differentiation proteins previously reported as present on the surface of human MSCs			
CD49a	ITGA1	BM mononuclear stem cell marker for multipotency	[25, 26]
CD49c	ITGA3	Potential predictive stem cell marker for BM-MSCs and AT-MSCs	[27, 28]
CD49d	ITGA4	AT-MSCs surface protein sensitive for cryopreservation	[30]
CD49e	ITGA5	Part of the expression profile of AT-MSCs	[28, 29]
CD56	NCAM1	Negative marker for AT-MSCs	[31]
CD109	CD109	Identified on MSCs derived from equine umbilical cord intervascular matrix	[32, 33]
CD142	F3	Stem cell surface protein, but not specific to MSCs	[34]
Identified cluster of differentiation proteins that have not previously been described in the literature in relation to AT-MSCs or BM-MSCs neither in human nor horse			
CD61	ITGB3	Have not previously been described in the literature in relation to AT-MSCs or BM-MSCs neither in human or horse	
CD91	LRP1	Have not previously been described in the literature in relation to AT-MSCs or BM-MSCs neither in human or horse	
CD228	MFI2	Have not previously been described in the literature in relation to AT-MSCs or BM-MSCs neither in human or horse	
CD315	PTGFRN	Have not previously described in the literature studied in relation to AT-MSCs or BM-MSCs neither in human or horse	

*AT-MSCs* adipose tissue-derived mesenchymal stromal cells, *BM-MSCs* bone marrow-derived mesenchymal stromal cells, *ISCT* International Society for Cellular Therapy

### The protein and gene expression pattern for selected MSCs markers

The protein and gene expression pattern was measured for the commonly used positive MSCs markers CD29, CD44, CD90, CD105, and CD166, and negative MSCs markers CD34, CD45, and CD79a.

**Fig. 1:**
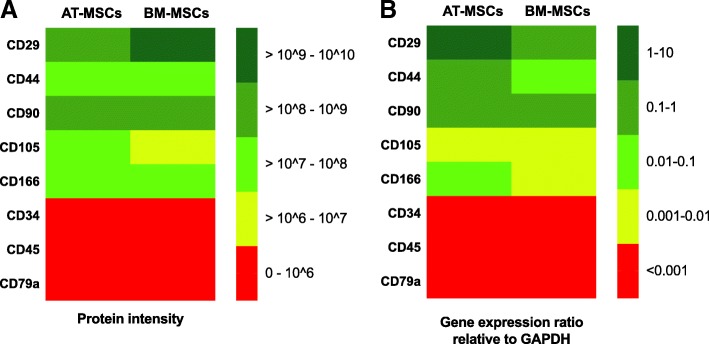
Heat maps showing the uniformity of the protein expression pattern (**a**) and gene expression pattern (**b**) in samples from equine adipose tissue-derived mesenchymal stromal cells (AT-MSCs) and bone marrow-derived mesenchymal stromal cells (BM-MSCs), for the commonly used positive mesenchymal stromal cell markers CD29, CD44, CD90, CD105, and CD166, and negative mesenchymal stromal cell markers CD34, CD45, and CD79a. The protein expression pattern is given by the mean of the label free quantification intensity measured in samples from three individuals. The gene expression pattern is given by the mean of the gene expression relative to GAPDH in samples from three individuals

The mean label-free quantification (LFQ) intensity was above 10^7^ for CD29, CD44, CD90, and CD166, with a higher mean LFQ intensity in samples from AT-MSCs compared to BM-MSCs, except for CD29 where the LFQ intensity was higher in the samples from BM-MSCs (Fig. 1a). The cell surface protein CD105 was identified in all AT-MSCs samples, but only in two of the three BM-MSCs samples. The LFQ intensity ranged from 4.2 × 10^6^–1.7 × 10^7^ (Fig. 1a). The cell surface proteins CD34, CD45, and CD79a were not identified in any of the samples (Fig. 1a).

The relative gene expression ratio for CD29, CD44, and CD90 ranged from 0.06 to 3.14, and for CD105 and CD166, the relative gene expression ratio ranged from 0.002 to 0.014 except for one AT-MSCs sample, where the relative gene expression ratio for CD166 was 0.15. The mean relative gene expression ratios for CD29, CD44, CD90, CD105, and CD166 were higher in AT-MSCs samples compared to BM-MSCs samples. The relative gene expression ratios for CD34, CD45, and CD79a were < 0.0003 in all AT-MSCs and BM-MSCs samples and > 0.05 in the positive control samples.

## Discussion

This is the first study in the horse, where the cell surface proteomes of AT-MSCs and BM-MSCs have been analyzed by use of biotinylation of the intact cells to enrich the plasma membrane proteins followed by MS for identification of the proteins with high confidence. This pipeline revealed identification of 1239 proteins including 19 CD markers.

Enrichment procedures like biotinylation aim to increase the relative abundance of proteins of interest, but all accessible proteins in the sample will be labeled with biotin, including intracellular proteins released due to cell death and extracellular proteins. However, the enrichment strategy in this study was considered successful, because the relative number of CD markers compared to the total number of identified proteins was markedly higher than the relative number of CD markers identified in a comparable MS study of equine umbilical cord MSCs without enrichment of the cell surface proteins [19]. Here, a total of 2118 proteins was identified of which only 14 proteins were assigned as CD markers [19].

The 19 identified CD markers included CD29, CD44, CD51, CD71, CD73, CD90, CD105, and CD166, which have also been identified as equine stem cell surface proteins in previous studies [5, 7, 19–21]. CD29, CD44, CD73, CD90, and CD105 are the five positive surface markers included in the ISCT criteria of human MSCs. However, in studies of equine stem cell surface proteins, only CD29 has been robustly positive in equine stem cells, while CD44, CD73, CD90, and CD105 have been identified with different expression patterns [5, 7, 19, 21, 22]. The inconsistent findings of CD105 on the surface of equine stem cells [7, 21, 22] were supported by the observations in this study, but the other four human ISCT-positive surface markers were identified with high confidence.

CD51 has been identified on the surface of MSCs derived from equine peripheral blood and AT [20] and CD71 in the proteome from MSCs derived from equine umbilical cord [19]. CD51 is known to be an endothelial cell marker and has been shown to be upregulated in mouse BM-MSCs during osteoblastic differentiation, suggesting a putative role in osteogenesis [20]. The role of CD51 and CD71 in equine MSCs is not clearly known, and further studies are required to establish them as stem cell markers. Stem cells should lack expression of CD45, CD34, and CD79a, and these proteins were not observed in the MS analysis of the enriched MSCs samples. Equine CD34 and CD45 have been identified in a previous comprehensive MS analysis of different equine tissues and body fluids [23], showing that the proteins can be identified using MS. Since CD34 and CD45 were not identified in this study, our findings support their capacity as true negative identifiers. To our knowledge, equine CD79a has never been identified by MS analysis. Identification of this protein in another cell type is needed to confirm that this is a true negative identifier for MSCs in MS studies.

Multiple factors have been suggested to explain the variable expression pattern of equine stem cell surface markers, for example differences in culture conditions and timeframes [5], distinct surface antigens for MSCs of different origin [22], and even individual differences between donors [22]. Another challenge often encountered in equine research is the lack of suitable antibodies of high quality and consistent performance across different laboratories [5, 7]. In this study, we employed two technologies that do not rely on antibodies as alternatives to immunophenotyping: MS to identify relevant proteins, and qPCR to identify relevant genes. Mass spectrometry has the advantage of identifying the markers at protein and peptide level being independent of protein antibodies for specific identification [23, 24]. However, this technology is still reserved for specialized proteomic core facilities, and its applicability for more general use is therefore limited. qPCR is a more commonly available technology. Radcliffe et al. [5] studied the temporal expression of CD29, CD44, CD90, CD11a/CD18, and CD45RB, both at the mRNA and protein level. They found that at all culture points tested, the gene mRNA expression followed the same pattern as the cellular protein expression. The uniformity between mRNA and protein expression patterns was supported by the observations of this study in terms of both expression pattern and positive and negative identifiers. Taken together, the results of these studies emphasize that gene expression analysis on mRNA might be of great value for identification of equine MSCs and as a method to verify findings on protein level, especially when suitable antibodies are lacking.

To our knowledge, this is the first study to identify CD49a, CD49c, CD49d, CD49e, CD56, CD61, CD91, CD109, CD142, CD166, CD228, and CD315 in equine MSCs. The majority of these proteins have been reported to be present on the surface of human AT-MSCs and BM-MSCs. In humans, CD49a proved useful as a positive marker for the most multipotent cells from a heterogeneous pool of BM mononuclear stem cells [25]. However, selection for CD49a-positive cells in a AT-MSCs population showed only a minor advantage to reduce heterogeneity [26]. In an attempt to identify chondrogenic potency predictors prior to chondrogenic differentiation of human BM-MSCs and AT-MSCs, it was demonstrated that CD49c was positively associated with GAG quantitation [27]. In another study of human BM-MSCs, the expression level of CD49c and CD49e was found to decrease after induction of chondrogenic differentiation in the presence of TGF-ß3 [28], which suggests that CD49c may be a predictive stem cell marker in humans. CD49e has also been identified as part of the expression profile of human AT-MSCs although less than 28% of the cells were positive for this marker [29]. The expression of CD49d on human AT-MSCs was found to decrease after cryopreservation and thawing even though the immunophenotypic marker expression was largely preserved, and their multipotency was maintained [30]. We also observed a lack of CD49d in AT-MSCs, but further investigation is needed to state if this is related to the phenotype. CD56 was observed in all AT-MSCs samples from horses and absent in all BM-MSCs samples. Human AT-MSCs have shown a variable positivity for CD56, and it is generally considered a negative marker for human AT-MSCs [31]. The diverging results could be due to different biological characteristics of this protein across species. In humans, CD109 and CD142 have been identified as stem cell surface proteins, but their presence has not been specific to the MSCs phenotype [32–34].

CD61, CD91, CD228, and CD315 have not previously been studied in relation to AT-MSCs and BM-MSCs either in human or horse. Further investigation and validation will be needed to test the immunological characterization of these proteins in the horse and determine their value as equine MSCs surface markers.

## Conclusions

In conclusion, the findings of this study show that enrichment of the MSCs surface proteome by biotinylation followed by MS analysis is a valuable alternative to immunophenotyping of surface markers, when suitable antibodies are not available. Furthermore, the method is very useful for mining of the cell surface proteome to identify potential additional equine stem cell markers. Furthermore, the results support using gene expression analysis to verify the data by another method, and as a valuable alternative to immunophenotyping for identification of specific MSCs markers.

## Abbreviations

AT: Adipose tissue
BM: Bone marrow
CD: Cluster of differentiation
DMEM: Dulbecco’s modified Eagle’s medium
EM: Expansion medium
FBS: Fetal bovine serum
ISCT: International Society for Cellular Therapy
LFQ: Label free quantification
MS: Mass spectrometry
MSCs: Mesenchymal stromal cells
TEAB: Triethylammonium bicarbonate buffer
